# Comparative genomic and phenotypic analysis of potential beneficial properties of *Bifidobacterium adolescentis*

**DOI:** 10.1128/msphere.00673-25

**Published:** 2025-11-04

**Authors:** Mallory J. Van Haute, Katherine Chacón-Vargas, Chloe M. Christensen, Shara R. P. Yumul, Fatimah F. Abdulaali, Andrew K. Benson, Robert Hutkins, Thomas A. Auchtung

**Affiliations:** 1 Synbiotic Health, Inc, Lincoln, Nebraska, USA; 2 Department of Food Science and Technology, University of Nebraska-Lincoln 14719, Lincoln, Nebraska, USA; 3 Nebraska Food for Health Center, University of Nebraska-Lincoln 14719, Lincoln, Nebraska, USA; University of Michigan Medical School, Ann Arbor, Michigan, USA

**Keywords:** *Bifidobacterium*, *adolescentis*, probiotic, beneficial, GABA, folate, β-galactosidase, lactose, resistant starch

## Abstract

Certain strains of *Bifidobacterium adolescentis* inhabiting the gastrointestinal tract have been shown to possess properties that are purported to improve host health. However, given the genetic and phenotypic diversity that has been reported among *B. adolescentis* strains, it is unclear if and to what extent these traits are conserved across the species. Accordingly, we examined potentially beneficial properties using a combination of genomic and *in vitro* approaches. Phylogeny inferred from core genomes of 148 *B. adolescentis* isolates revealed five major lineages. At the functional genomic level, the strains were separated into three groups based on carbohydrate-active enzymes (CAZymes) that were predicted to act on a diverse range of substrates. Fifteen different *B. adolescentis* strains representing the major phylogenetic groups were tested *in vitro* for several metabolic traits. The ability to metabolize lactose (all strains; 2–232 β-galactosidase Miller units) and resistant starch (66% of strains; 26–74% utilization) and to produce γ-aminobutyric acid (80% of strains; 0.3–14.4 mM) and folate (all strains; 23–281 ng/mL) was common but showed substantial quantitative variation across strains under the conditions tested. Genes for the formation of antimicrobial compounds (7% of genomes) and antibiotic resistance (23% of genomes) were less frequent. While individual traits did not exhibit strong phylogenetic clustering, *B. adolescentis* strains had higher CAZyme gene counts relative to other comparison species, suggesting a broader adaptation for carbohydrate metabolism. Further studies on the distribution of beneficial traits and their genetic basis will provide insights into the contributions of *B. adolescentis* to health.

**IMPORTANCE:**

*Bifidobacterium adolescentis* is a gut commensal that is prevalent among healthy adults and centenarian populations, potentially contributing to host health through diverse functional properties. Here, through genomic and phenotypic analyses, we advanced our understanding of the prevalence of multiple potentially beneficial properties of *B. adolescentis,* including those associated with improving lactose tolerance, metabolic health, and mood, and supplying vitamins and inhibiting pathogens. Our findings revealed substantial quantitative variation in metabolic activities and production of relevant end-products across strains, highlighting the importance of strain-level differences and the health benefits they may confer. In addition, while the presence of specific genes was partially predictive of the magnitude of traits, the associations between genetics and phenotypes established here provide a foundation for improving future predictions.

## INTRODUCTION

Bifidobacteria inhabiting the gastrointestinal tracts of animals are well known for properties that benefit their host, including the ability to produce short-chain fatty acids (1, 2), enhance gut barrier function (3, 4), protect the host against infection (5, 6), and modulate the host’s immune responses (7). The species *Bifidobacterium adolescentis* is of particular interest due to its high prevalence in the healthy adult microbiota (8) and its ability to utilize a wide range of carbohydrates (9–12) and produce bioactive molecules (13, 14). In addition, a reduction in *B. adolescentis* has been reported for several diseases (15), including COVID-19 (16) and some cancers (17).

Although *B. adolescentis* is generally found at relatively low abundances early in life (18), this species, along with *Bifidobacterium longum* subsp. *longum* and *Bifidobacterium pseudocatenulatum*, is one of the most abundant and prevalent *Bifidobacterium* species in adulthood (19). Multiple studies have shown that this species is among a consortium of bifidobacteria associated with healthy aging and longevity (20–23).

While many bifidobacteria can utilize oligosaccharides, including those derived from milk (24), strains of *B. adolescentis* are capable of metabolizing a wide range of plant oligo- and polysaccharides (8, 12), including xylooligosaccharides (XOS) (25), inulin (26), and mannan oligosaccharides (27). Some *B. adolescentis* strains also metabolize resistant starch (28), and this has been linked with improved metabolic health (29, 30). The intestinal abundance of *B. adolescentis* has additionally been correlated with consumption of certain foods (31), such as wheat (32) and milk (33). *B. adolescentis* is adept at metabolizing lactose (high in milk) and other galactosides, such that their high prevalence and abundance among non-lactase-persisting adults has led researchers to suggest that *B. adolescentis* (among other bifidobacteria) is responsible for lactose tolerance among these individuals (34).

*B. adolescentis* also produces several bioactive compounds, including γ-aminobutyric acid (GABA), folate, and antimicrobials. GABA is a mammalian inhibitory neurotransmitter that is produced not only by the host, but also by some members of the gut microbiome, including *B. adolescentis* (13). While bacteria can produce GABA to raise intracellular pH, they may also influence host physiology, including brain activity, mood, and sleep (35). A glutamate decarboxylase and transporter, encoded by *gadB* and *gadC*, respectively, are key for GABA production in many bacteria (36). Additionally, folate is an essential vitamin (B9) for humans and animals and must be obtained from either dietary sources or gastrointestinal bacteria that synthesize folate *de novo* (14, 37). Folate is required for many metabolic pathways, and its deficiency has been linked with disorders of multiple organs and some cancers (38). In addition to GABA and folate, antimicrobial compounds (bacteriocins and other secondary metabolites) may also be produced (39–41), although they have not been well studied in bifidobacteria.

Previous genomic analyses have shown that *B. adolescentis* does not have separate subspecies and has some of the highest genetic diversity of any *Bifidobacterium* lineage (12, 42, 43). With the increased availability of high-quality *B. adolescentis* genomes (8, 44–46), we aimed to better define the beneficial traits of this species by examining both genomic and phenotypic properties. Using existing databases, we examined their abundance across the lifespan of healthy people, compared the relevant genes in publicly available and newly isolated *B. adolescentis*, and investigated traits experimentally across a phylogenetically diverse selection of strains. Our findings suggest that while many of these properties are widespread across *B. adolescentis*, there is significant variation in their magnitude.

## RESULTS

### Metagenomic abundance of *B. adolescentis* over the human lifespan

**Fig 1:**
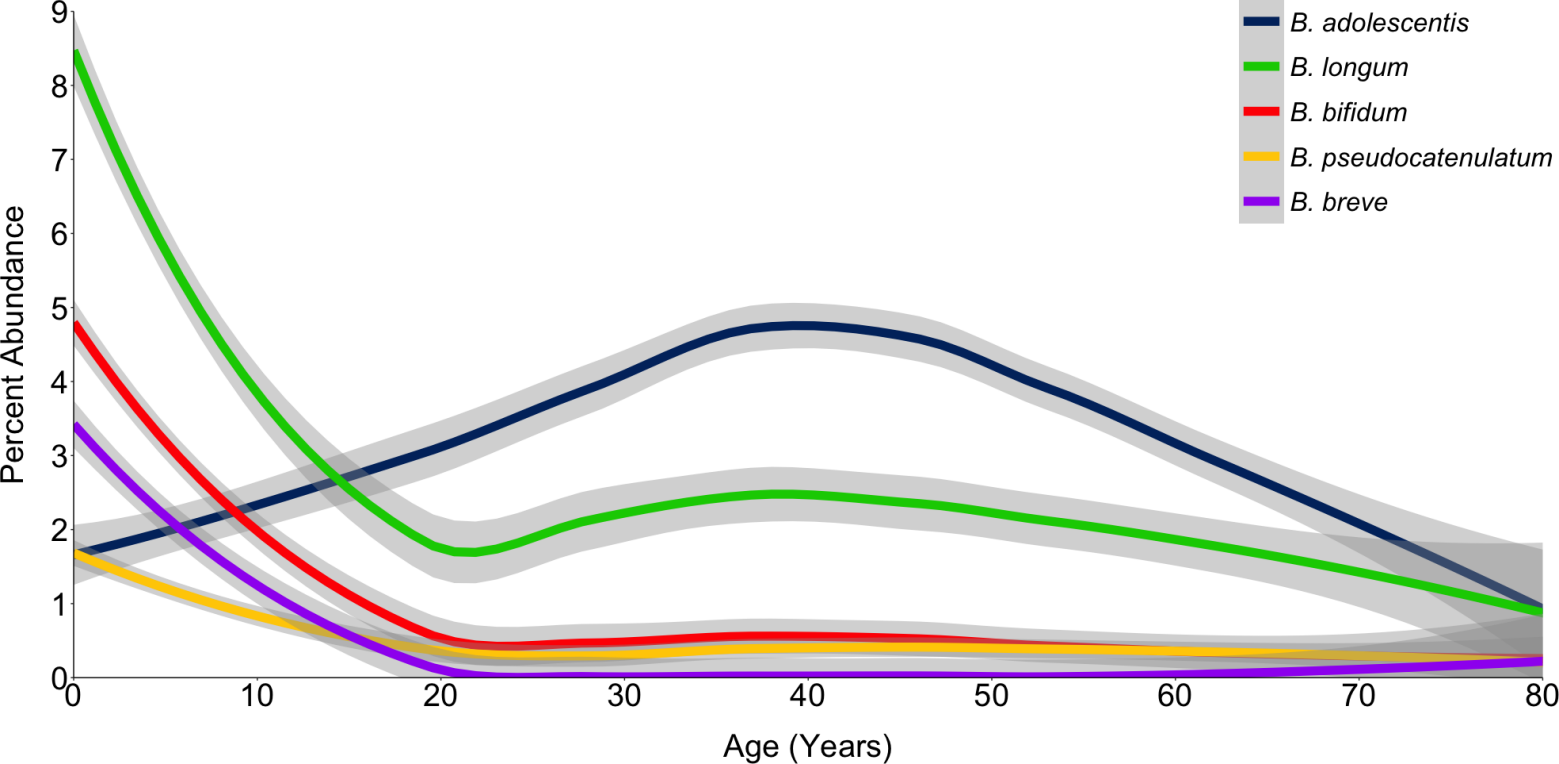
Abundance of *Bifidobacterium* in healthy individuals by age. LOESS fit for abundance of the five most common *Bifidobacterium* species in a curated database of shotgun metagenomic sequences from stool samples (*n* = 5,966 samples). Shaded regions represent 95% confidence intervals. Full data are listed in Table S1.

The association between *B. adolescentis* and health was first investigated by examining levels of the most abundant *Bifidobacterium* species in healthy people from an extensive database of diverse curated metagenomes (47). While *B. longum* was the most abundant *Bifidobacterium* present early in life, the relative abundance of *B. adolescentis* increased through childhood, and by age 15 became the most abundant *Bifidobacterium* species (Fig. 1; Table S1). Abundance of *B. adolescentis* increased to nearly 5% of the fecal microbiome at age 40 before decreasing approximately 1% per decade thereafter. By contrast, in unhealthy people, the abundance of all *Bifidobacterium* species was below 2% at all ages (Fig. S1).

### Compilation of *B. adolescentis* genome sequences and isolates

To compare properties of *B. adolescentis*, we first gathered publicly available genome data. One hundred thirty-seven high-quality non-redundant *B. adolescentis* genomes were available from NCBI for our subsequent analyses (Table S2). While the presence of *B. adolescentis* has been reported among cows, pigs, rabbits, elephants, and nonhuman primates (48–50), only one public genome of non-human origin was available (cow rumen strain LMG 11579; GCF 002108155.1), and none in our collection. To analyze the genomic and *in vitro* properties of as diverse a collection of *B. adolescentis* strains as possible, we attempted to isolate *B. adolescentis* from animals and humans, as well as from publicly available and commercial probiotic products.

Fecal samples obtained from healthy humans and wild, domesticated, and zoo animals were plated on *Bifidobacterium*-selective iodoacetate mupirocin (BSIM) and other media. Although numerous isolates of *B. adolescentis* were obtained from humans (*n* = 14 donors), the only animal which *B. adolescentis* was successfully isolated from was a cotton-top tamarin (*Saguinus oedipus*) (Table S3).

We also acquired commercial and publicly available *B. adolescentis* strains (Table S4). These included iVS-1, PRL2019, ATCC 15703 (the type strain), and the previously described infant strain, L2-32 (2). Efforts to isolate *B. adolescentis* from other commercial products that claimed to contain this species were unsuccessful, though qPCR using species-specific primers showed that *B. adolescentis* was present at low levels in all commercial products from which it was not isolated (0.0001–0.09% of cells).

To have diverse phylogenetic representation, yet a manageable number of strains to test, 15 *B. adolescentis* strains were selected based on rRNA operon internal transcribed spacer sequence diversity and having been previously studied. Isolates without a publicly available genome sequence were sequenced and their genomes were assembled.

**Fig 2:**
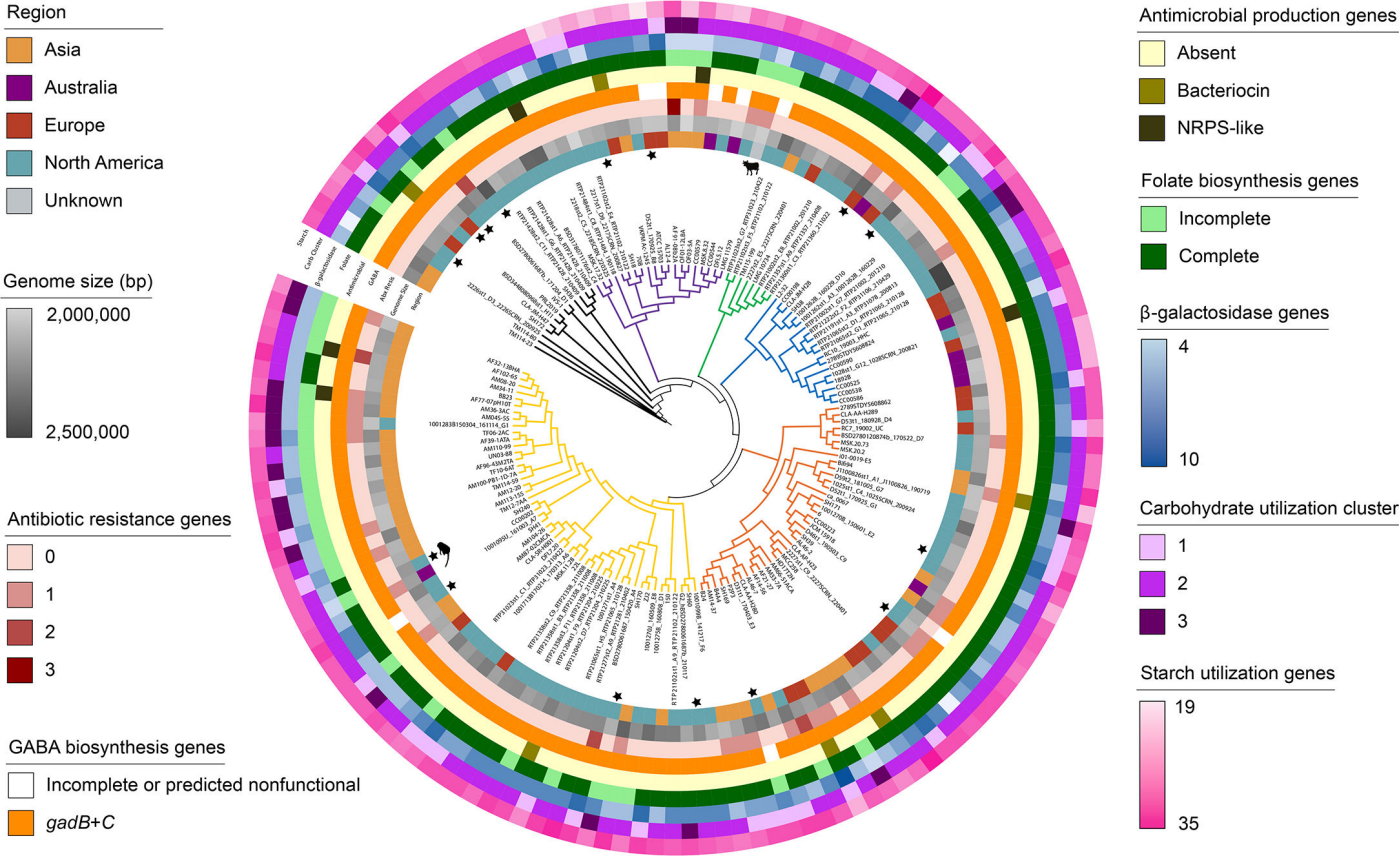
Genomic diversity and beneficial traits of *B. adolescentis*. Phylogenetic tree showing the relationship of 148 *B. adolescentis* genomes. The five clades that include most of the strains are colored. The tree was predicted in Orthofinder with MAFFT and FastTree analysis of 1,117 single-copy orthogroups, then visualized with iTOL. From inside to outside, the rings are colored by (i) geographic region where the strains were isolated, (ii) genome size, (iii) antibiotic resistance genes, (iv) GABA genes, (v) genes for antimicrobial compound production, (vi) genes for folate biosynthesis, (vii) β-galactosidase gene copy number, (viii) carbohydrate utilization cluster, and (ix) starch utilization genes. Stars indicate which strains were used for *in vitro* laboratory analyses and animal silhouettes denote non-human isolates.

The total number of *B. adolescentis* genomes obtained for characterization in this study was 148, with all but two being of human origin (Fig. 2). Phylogenetic analysis of 1,117 single-copy orthogroups in Orthofinder using MAFFT and FastTree showed that after 13 strains at the root of the tree, the remaining 135 sequences clustered into one of five distinct lineages. There did not appear to be correlations between phylogeny and geographic origin (over four different continents), genome size (2,037,347–2,499,890 bp), or the other traits studied. In addition, the two strains isolated from non-human sources were not closely related.

Fifteen *B. adolescentis* strains representing diverse phylogenetic groupings were subsequently selected for *in vitro* analyses of metabolic properties. We also obtained and tested non-*B*. *adolescentis* strains that have been previously studied for beneficial effects and are commonly sold in the US probiotic market (Table S5) (51–54). These 16 comparator strains were from the genera *Bifidobacterium*, *Heyndrickxia*, *Lactobacillus*, *Lacticaseibacillus*, *Lactiplantibacillus*, *Limosilactobacillus*, *Lactococcus*, *Pediococcus*, and *Streptococcus*.

Antimicrobial and safety-related genes were analyzed computationally (Supplemental Text; Fig. 2). Only 6 out of 148 *B. adolescentis* genomes had genes predicted to encode a bacteriocin, while 5 other genomes were predicted to contain genes for non-ribosomal peptide synthetase-like enzymes. Since none of the 15 strains we possessed were predicted to contain antimicrobial genes, we did not assess their activity *in vitro*. Genes for virulence factors were not detected in the *B. adolescentis* genomes; however, at least one antibiotic resistance gene was predicted in 34 out of 148 (23%) strains.

### Genomic potential for carbohydrate utilization

**Fig 3:**
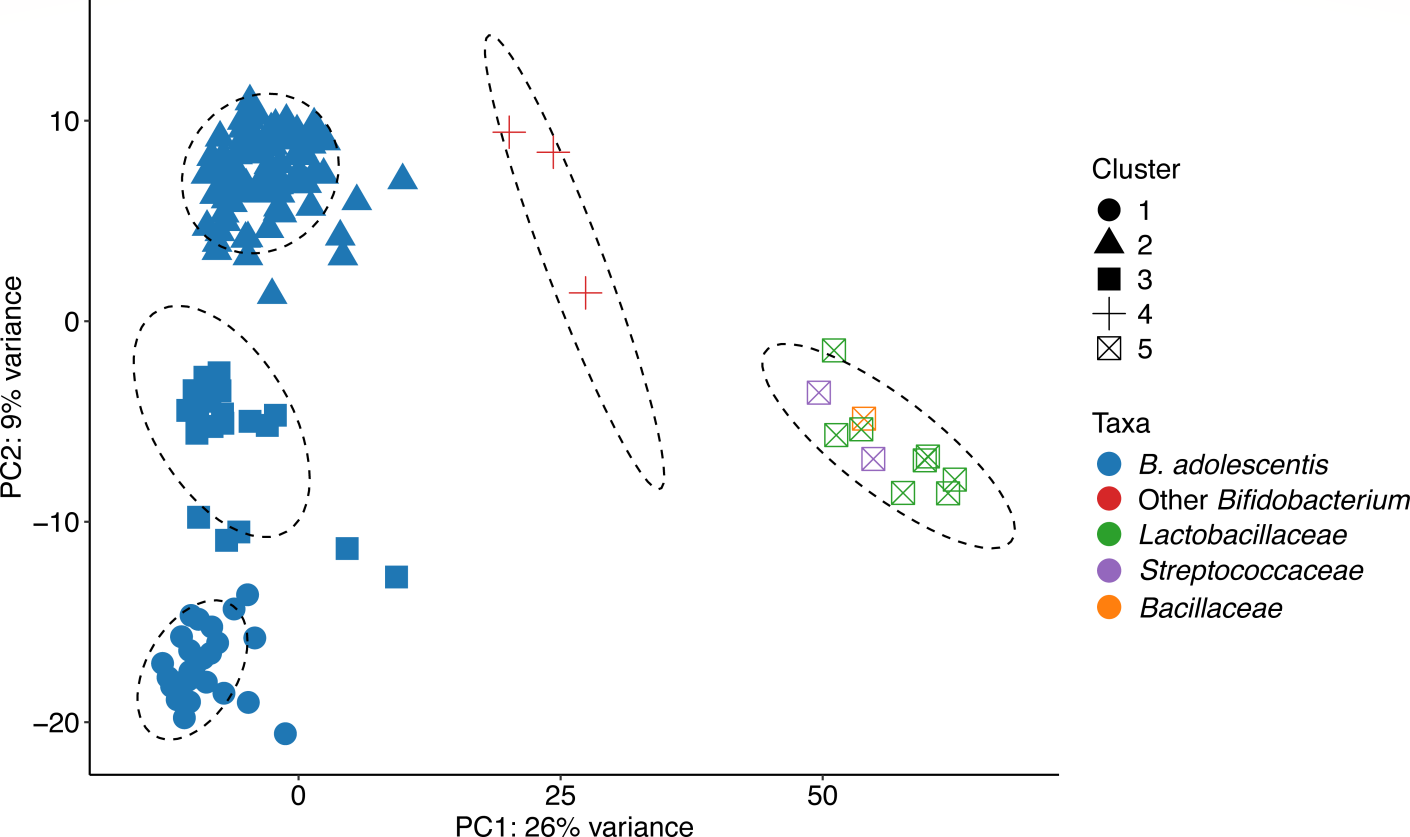
CAZyme profile of *B. adolescentis* and comparator strains. Principal component analysis of CAZyme profiles from 148 *B. adolescentis* and 16 comparator strains. Shapes denote the five *k*-means clusters. Ellipses represent a multivariate *t*-distribution at a 95% confidence interval. Full data are listed in Table S7.

Analysis of carbohydrate-active enzymes (CAZymes) confirmed the strong carbohydrate-degrading potential of *B. adolescentis*, highlighting possible competitive advantages in starch-rich niches and its capacity for lactose utilization. Annotation showed 145 CAZyme families within *B. adolescentis* genomes (Table S6), compared to 239 CAZyme families in comparator strains. K-means clustering of CAZyme profiles divided all strains into five groups (Fig. 2 and 3; Table S7). Differences in copy numbers of glycoside hydrolase (GH) families 1, 2, and 13, and carbohydrate esterase families 2 and 20 explain the separation of *B. adolescentis* into three distinct groups (Fig. S2; Table S8). Compared to non-*Bifidobacterium* strains, *B. adolescentis* exhibited higher copy numbers of GH2, 3, 5, 12, 13, 36, 42, 43, 77, and 172 and glycosyl transferase family 51, with reduced abundance of GH1. Furthermore, predicted CAZyme gene clusters (CGCs) for starch and arabinoxylan utilization were widespread in *B. adolescentis* (mean 3.5 and 2.0, respectively) but sparse in the comparator strains (Fig. S3; Table S9). In contrast, CGCs for metabolism of cellobiose and human milk oligosaccharide components were present in multiple comparator strains, but were absent in *B. adolescentis*. Neither CAZyme families nor CGCs corresponded to phylogeny.

### Carbohydrate utilization

**Fig 4:**
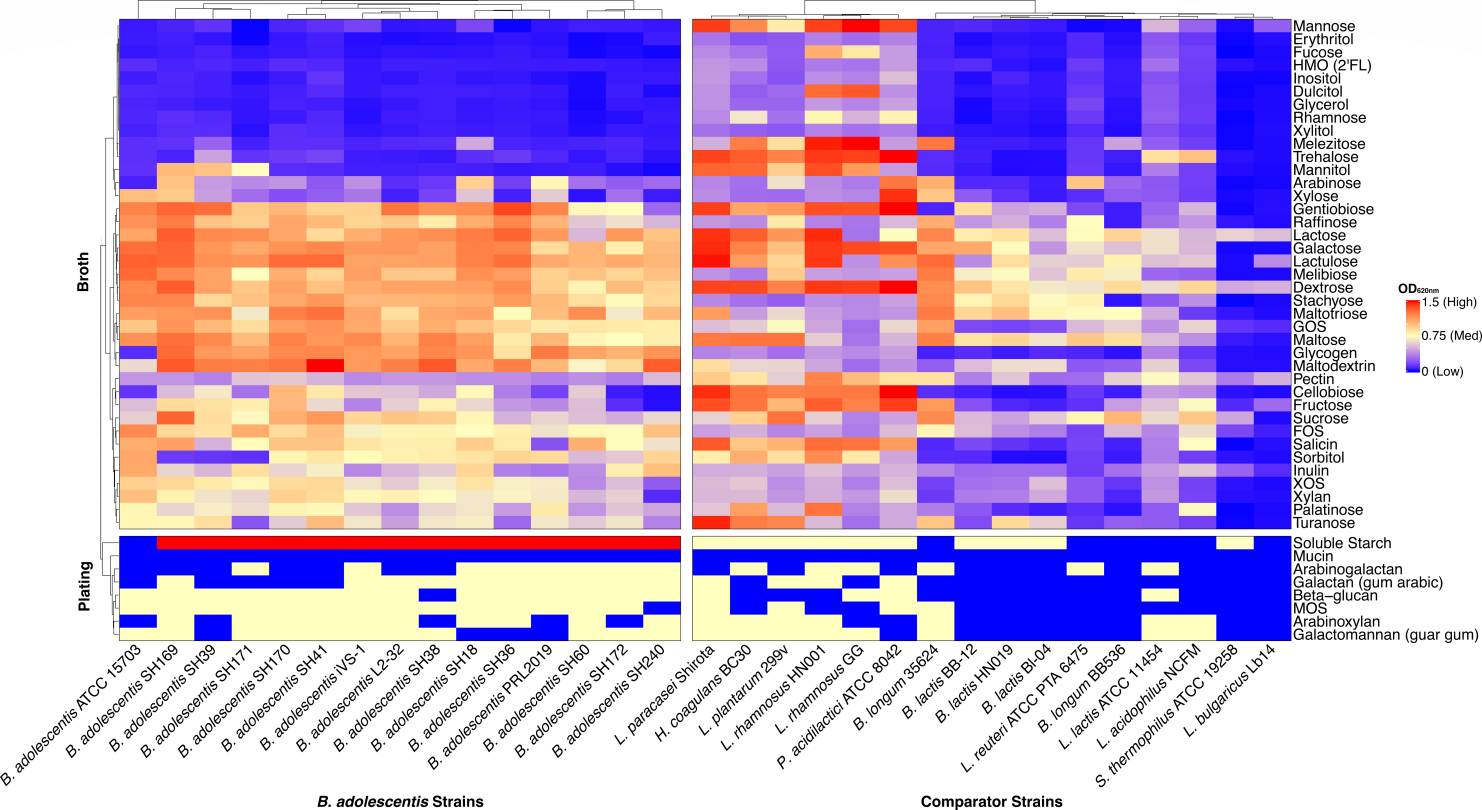
Carbohydrate utilization of *B. adolescentis* and comparator strains. The maximum optical density at 620 nm in liquid MRSc of 39 individual carbohydrates over 72 h growth (top), and level of growth on MRSc agar plates containing one of eight individual carbohydrates (bottom). Full data and taxonomic names are listed in Table S10.

Carbohydrate utilization among the strains was assessed by measuring the maximum optical density of cultures grown on 39 different substrates in liquid media. An additional eight substates were opaque in broth and therefore growth was measured on agar plates (Fig. 4; Table S10). The *B. adolescentis* strains metabolized a broad range of carbohydrates, with 26–34 carbohydrates (mean 29.5) supporting growth of individual strains (OD_620_ > 0.4, or more growth on plates than with no carbohydrate added). By comparison, the comparator strains generally utilized fewer carbohydrates (between 3 and 39; mean: 21.9). All 15 *B. adolescentis* strains were able to grow on raffinose, lactose, galactooligosaccharides (GOS), and fructooligosaccharides (FOS), and many could utilize sorbitol (12 strains), inulin (11 strains), or XOS (13 strains). However, few *B. adolescentis* strains could metabolize other sugar alcohols, such as mannitol (3 strains), and none could metabolize mucin, the human milk oligosaccharide, 2′-FL, or the monosaccharides mannose or fucose. In addition, *in vitro* growth generally corresponded to activity predicted by analysis of CGCs. For example, growth was more common on soluble starch (14/15 *B. adolescentis* vs 10/16 comparators) and arabinoxylan (10/15 *B. adolescentis* vs 7/16 comparators), for which the CGC are more frequent in *B. adolescentis* than comparator strains.

### Resistant starch utilization

**Fig 5:**
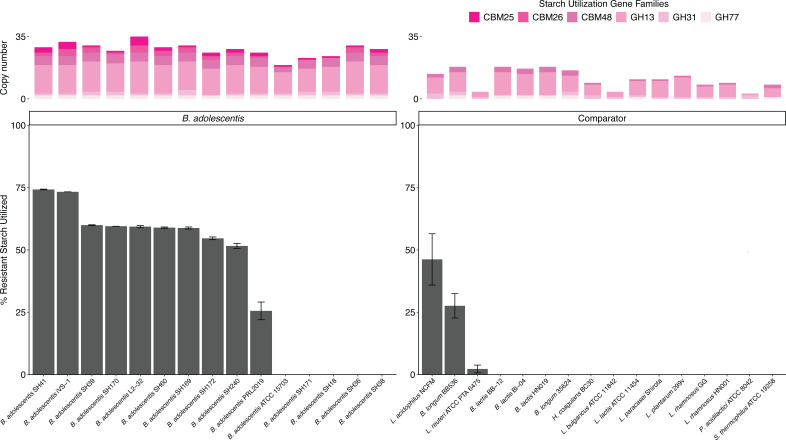
Resistant starch utilization in *B. adolescentis* and comparator strains. (Top) Bars denote copy number of genes related to starch metabolism in the genome of each strain. CBM, carbohydrate-binding module; GH, glycoside hydrolase. (Bottom) Resistant starch utilization by *B. adolescentis* and comparator strains grown in phosphate-buffered MRS (no sugar) + 1% potato starch. Full data and taxonomic names are listed in Tables S11 and S12.

Genes from CAZyme families with potential amylase activity or starch binding were examined. All *B. adolescentis* genomes contained between 19 and 35 genes (mean: 28.2) predicted to encode for starch utilization, compared to only 3–18 genes in other species (mean: 11.3) (Fig. 2 and 5; Table S11). These belonged to GH families 13, 31, and 77 and carbohydrate binding modules (CBM) 25, 26, and 48. To assess *in vitro* capacity of *B. adolescentis* strains to metabolize resistant starch, the amount of resistant potato starch (type 2) remaining in liquid culture after 48 h of growth was quantified (Fig. 5; Table S12). Ten of the 15 *B. adolescentis* strains metabolized resistant starch (26–74% utilization), whereas only two comparator strains substantially reduced the amount of resistant starch (*L. acidophilus* NCFM, 46% utilization and *B. longum* BB536, 28% utilization). None of the starch-utilization enzyme families were consistently different between resistant starch and non-resistant starch utilizing *B. adolescentis*, although the three strains with the lowest total number of starch-related genes exhibited no detectable resistant starch utilization.

### β-Galactosidase genetic capacity and activity

**Fig 6:**
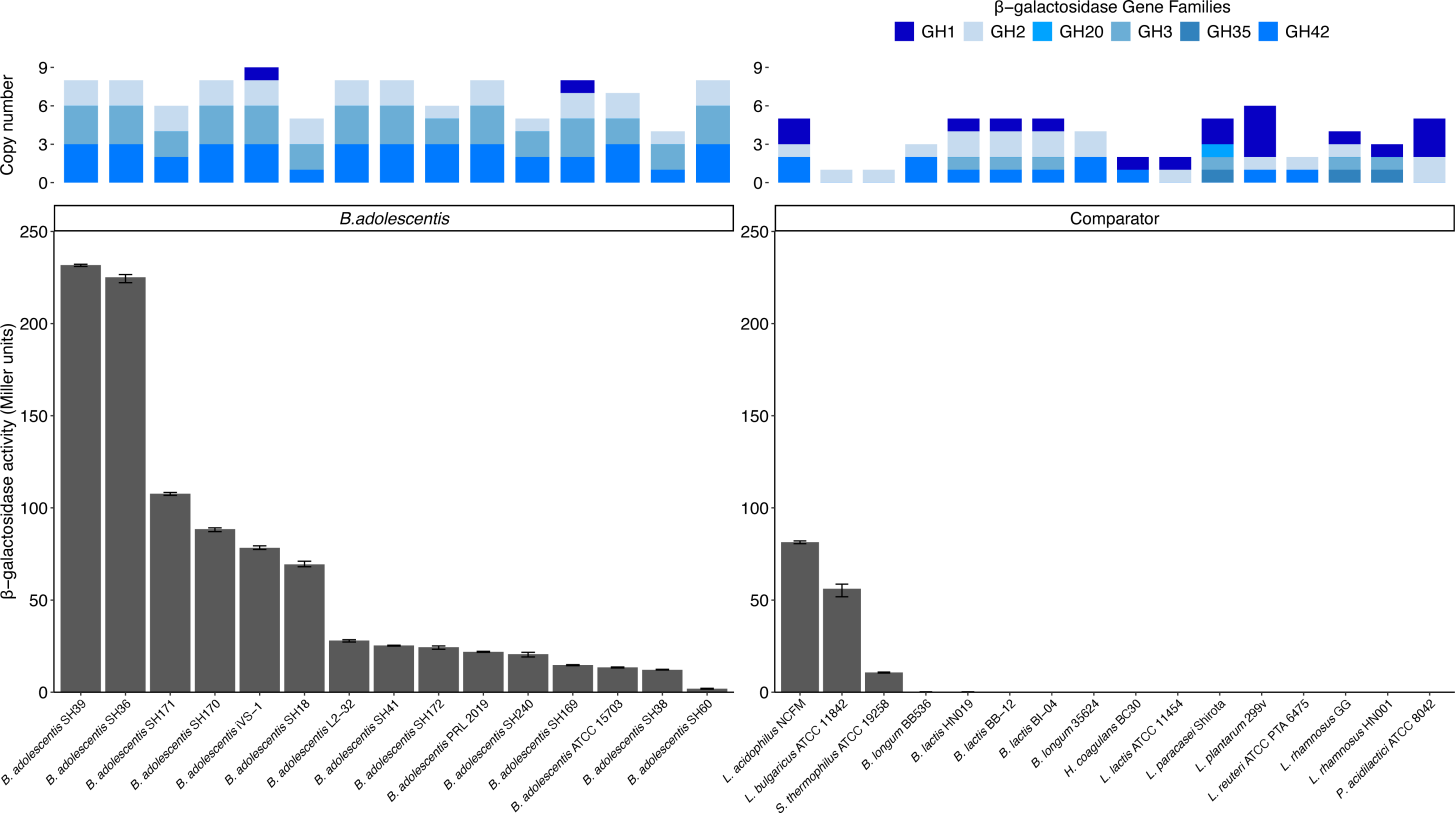
β-Galactosidase activity in *B. adolescentis* and comparator strains. (Top) Comparison of β-galactosidase genes by glycoside hydrolase family designation, predicted using dbCAN3. Colors represent different GH families. (Bottom) Comparison of whole-cell β-galactosidase activities. The bacteria were grown in Complex Gut Media + MOPS (CGMM) + 1% lactose at 37°C for 6 h. The β-galactosidase activity was determined by reaction with ONPG for 5 min, centrifuging 3 min, then addition of sodium carbonate to stop reactions. Absorbance was quantified at 420 and 550 nm, and activity was normalized to the number of cells, as enumerated by dilution plating. Data are in Tables S13 and S14.

Genes from GH families 1, 2, 3, 20, 35, and 42 that may include enzymes with β-galactosidase activity were examined in the 148 genomes. All *B. adolescentis* strains contained β-galactosidase genes, ranging from 4 to 10 copies (mean: 7.1) (Fig. 2 and 6; Table S13). By comparison, the non-*B*. *adolescentis* strains contained between 1 and 6 copies (mean: 3.6). Most *B. adolescentis* strains not only contained more copies than the comparator strains but also carried more than representatives of other *Bifidobacterium* species (1–11 copies, mean: 5.3).

We also investigated β-galactosidase activity *in vitro*. Strains were grown in complex gut media + MOPS (CGMM) containing lactose, then whole cells (Fig. 6; Table S14) and lysed cells (Table S14) were analyzed for enzymatic activity. *B. adolescentis* strains had a wide range of activity, varying from 2 to 231 Miller units (Mu) for whole cells (mean: 64 Mu), and 2–207 Mu for lysed cells (mean 50 Mu). *B. adolescentis* strains generally had higher activity than the comparator strains (whole cells, 0–81 Mu, mean 9 Mu; lysed cells, 0–109 Mu, mean 15 Mu), as many of the comparator strains had no detectable β-galactosidase activity under the conditions tested. There was also no apparent correlation between the number of β-galactosidase gene copies and activity (whole cells, *r* = 0.20; lysed cells, *r* = 0.35; Table S15).

As *B. adolescentis* has been reported to reduce gas formation during *in vitro* fecal fermentation of lactose (10), we next examined whether strains with higher β-galactosidase activity would be more effective at reducing gas formation by fecal microbiomes than those with lower β-galactosidase activity. Gas production was reduced 25–79% by all of the tested *B. adolescentis* strains (mean: 62% reduction) (Fig. S4; Table S16). Overall, however, β-galactosidase activity of *B. adolescentis* strains did not correlate with gas reduction (*r* = 0.22 and 0.17 for whole and lysed cells; Table S15), although many strains with low or no β-galactosidase activity did not significantly reduce gas.

### Genetic capacity and production of GABA

**Fig 7:**
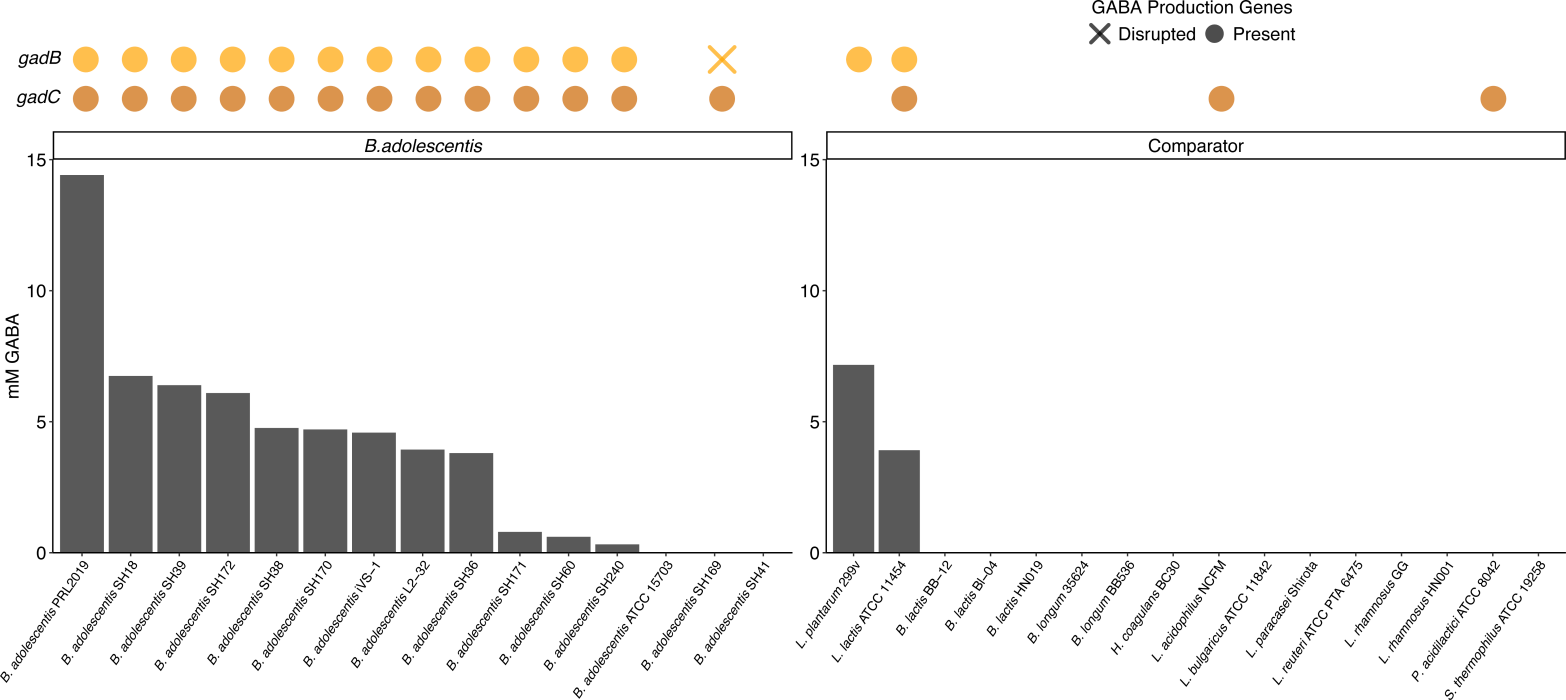
GABA production in *B. adolescentis* and comparator strains. (Top) The presence of complete (circles) or disrupted (cross) GABA production genes (*gadB* and *gadC*) in the genome of each strain. (Bottom) GABA production by *B. adolescentis* and comparator strains grown for 48 h in pH 5.6 MRS + 1% glutamic acid. Full data and taxonomic names are listed in Tables S17 and S18.

*B. adolescentis* genomes were analyzed to determine the presence of *gadB* and *gadC*, genes thought necessary for the conversion of glutamate to GABA. Of the 148 strains, 142 had at least one copy of both genes for GABA production, while 1 strain had only *gadB*, and 5 strains did not harbor either gene (Fig. 2 and 7; Table S17). Among the other genomes examined, only *Lactococcus lactis* ATCC 11454 contained complete GABA production genes. Notably, the absence of these genes in some genomes may be due to incomplete assemblies or improper annotations from the draft genomes available (95–100% complete).

When *in vitro* production of GABA was measured, 12 out of 15 *B. adolescentis* strains produced 0.3–14.4 mM GABA (mean: 4.8 mM; Fig. 7; Table S18). Two of the five strains that did not appear to contain either of the two *gad* genes (ATCC 15703 and SH41) were unable to produce GABA. In addition, strain SH169 possessed GABA production genes, but *gadB* was split into two pseudogenes (*gadB1* and *gadB2*; Fig. S5), and the strain did not produce GABA. The *B. adolescentis* SH169 *gadB1* encodes a protein 100% identical to the N-terminal 145 amino acids of *gadB* from iVS-1 (representative of typical gene arrangement), while the coding region of SH169 *gadB2* overlaps that of *gadB1* by 25 nt and encodes a protein with just one amino acid difference to the last 255 amino acids of iVS-1 *gadB*. In addition, SH169 also has a short insertion of 216 nt between *gadB* and *gadC* that encodes a hypothetical protein of 71 amino acids, suggesting this region has undergone significant genomic events in this strain. Interestingly, one of the other 147 *B. adolescentis* strains, D46t1_190503_C9, had the same altered genetic structure in this region, suggesting a common origin. However, while these strains are in the same lineage, they are phylogenetically distinct, suggesting the event was not ancestral. Another strain, BB23, also possessed only the short insertion (Fig. S5) and was found in a separate lineage. Furthermore, of the 16 non-*B*. *adolescentis* strains tested *in vitro*, only two produced GABA: 3.9 mM (*L. lactis* ATCC 11454) and 7.2 mM (*L. plantarum* 299v). *L. lactis* ATCC 11454 contains both *gadB* and *gadC*, while *L. plantarum* 299v only contains *gadB*, as has been previously documented (55).

### Genetic capacity and production of folate

**Fig 8:**
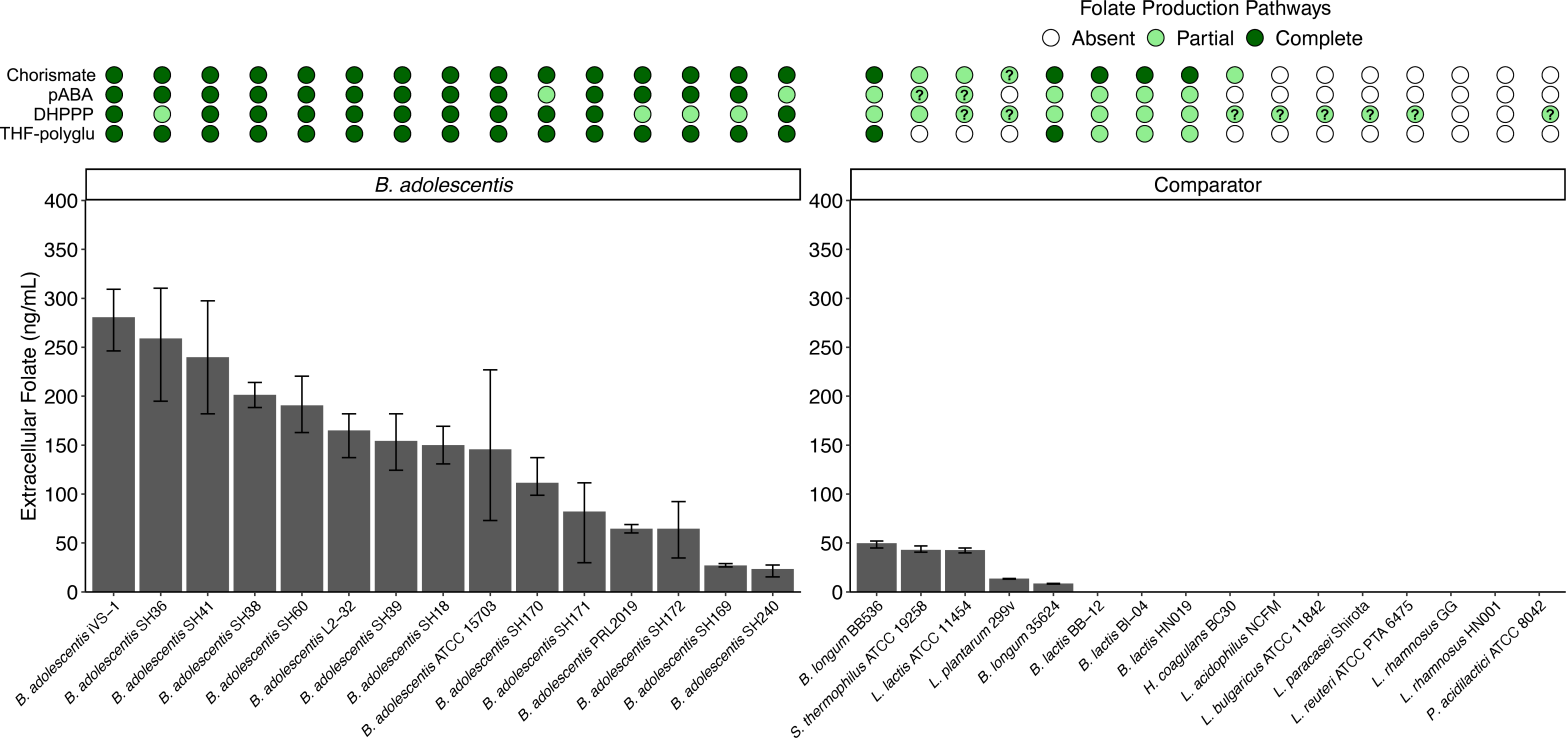
*In vitro* extracellular folate production in *B. adolescentis* and comparator strains. (Top) Circles denote completeness of folate metabolism pathways for production of chorismate (six genes), para-aminobenzoic acid (pABA; two genes), 6-hydroxymethyl-7,8-dihydropterin pyrophosphate (DHPPP; four genes), and tetrahydrofolate (THF)-polyglutamate (three genes), colored as absent (white), partial (light green), or complete (dark green) in the genome of each strain. Pathways with homologous genes of low identity (30–40%) are denoted with a question mark. (Bottom) Extracellular folate concentrations in supernatants of *B. adolescentis* and comparator strains grown for 24 h in MRSc, as measured via bioassay. Bars extend to the mean and whiskers span the 95% confidence interval. Full data and taxonomic names are listed in Tables S19 and S20.

All genes associated with folate production were analyzed, including those for producing chorismate (six genes), para-aminobenzoic acid (pABA; two genes), 6-hydroxymethyl-7,8-dihydropterin pyrophosphate (DHPPP; four genes), and tetrahydrofolate (THF)-polyglutamate (three genes) (Fig. 8; Table S19). Most *B. adolescentis* (109/148 strains) possessed these genes (>40% identity to reference *B. adolescentis* genes), although 25 strains were missing *pabC*, 12 strains were missing DHPPP gene 3.1.3.1, one strain was missing *folB*/*K*, and one strain was missing *folC*. In contrast, no comparator strains possessed all the known genes encoding for folate biosynthesis, and many lacked all known pathways. *In vitro* folate production was also examined (Fig. 8; Table S20). All 15 *B. adolescentis* strains produced extracellular folate (23–281 ng/mL, mean: 144 ng/mL). While five out of six *B. adolescentis* strains that did not possess all the genes were on the low end for folate production (23–112 ng/mL), the remaining strain did produce a high level of folate (259 ng/mL). Only five comparator strains produced measurable folate (8.5–50 ng/mL); although few genes for folate production were detected in three of these strains, there may be other pathways not present in bifidobacteria (56).

## DISCUSSION

Despite numerous reports suggesting that strains of *B. adolescentis* possess several beneficial traits (10, 13, 15, 30, 57), it was unclear how widespread these traits were across the species. Here, we examined the phylogeny and genetic and phenotypic properties of a diverse set of *B. adolescentis* strains representing human and animal isolates, either newly isolated or obtained from public databases or commercial products. Most of the 148 available strains of *B. adolescentis* were clustered into five major phylogenetic clusters with a high level of genetic diversity, in agreement with what has been previously observed (12, 42, 43). Additionally, these clusters did not correspond with continent of isolation, genome size, or the presence of beneficial traits (Fig. 2). This suggests that *B. adolescentis* strains have functionally diverse adaptations due to host-to-host transmission, dietary influences, and microbiome plasticity, as observed in other *Bifidobacterium* species (8, 12, 58). Genomic analyses and *in vitro* tests showed that β-galactosidase activity (100%; 2–232 Mu), resistant starch metabolism (66%; 26–74% utilization), and production of GABA (80%; 0.3–14.4 mM) and folate (100%; 23–281 ng/mL) were generally conserved traits, but of variable magnitude in *B. adolescentis* isolates. Notably, on average, *B. adolescentis* strains had higher β-galactosidase activity, resistant starch metabolism, and GABA and folate production *in vitro* than the comparator species tested, suggesting that *B. adolescentis* is a promising candidate for these areas of health enhancement than other species that are more commonly studied and consumed.

For lactose metabolism, strains were predicted to possess between 4 and 10 β-galactosidase genes, but this did not correspond to enzymatic activity (very little [1 strain], low [8 strains], medium [4 strains], or high [2 strains] levels) or reduction of gas from lactose during *in vitro* fecal fermentations. For some strains, this may be due to experimental conditions not optimized for expression of these enzymes. Likewise, some non-*B*. *adolescentis* strains with known activity in dairy products (59) also did not have expected activity. This could be due to unfavorable experimental conditions for expression such as being grown in CGMM instead of a rich fermentation medium more suitable for dairy applications. Another factor to consider for understanding lactose metabolism is the ecology of the strains and how they interact within the gut microbiome. Cross-feeding with *B. adolescentis* has been well documented (2). Whereas partnership with *Faecalibacterium* may be advantageous for digestive wellness (60), associating with *Megasphaera* (61, 62) may contribute to unwanted gas production.

*B. adolescentis* is one of the few primary degraders of resistant starch (63). All 148 *B. adolescentis* genomes were predicted to contain multiple starch gene clusters (up to 6 clusters), and more starch metabolism-associated genes in total (19–34 genes) than non-*B*. *adolescentis* comparator strains. Multiple species of *Bifidobacterium* are known to metabolize non-resistant starch (64–66). Yet for both forms of starch, the specific role of associated gene clusters is often unclear (11), and the ability to metabolize the structure depends on the presence of relevant binding modules, and for resistant starch, the type and source of the substrate (63). Here, we observed that most *B. adolescentis* strains could either grow well (51–75% degradation; nine strains) or not at all (five strains) on type 2 resistant starch derived from potatoes. Further comparative genomics and laboratory experiments would be valuable to explain the role of the different genes.

For GABA, most (96%) of the 148 strains we examined had both genes required for production (*gadB* and *gadC*), which is consistent with previous reports (13, 36). Strains tested *in vitro* produced either none (3 strains), low (3 strains), medium (8 strains), or high (1 strain) amounts. However, one strain contained both genes but did not produce measurable GABA under the conditions tested. It is possible that the inability of this strain to synthesize GABA was due to having a split *gadB* or a region inserted between *gadB* and *gadC*. In contrast, enhanced GABA production has been observed in recombinant strains of *B. adolescentis* where expression of the *gad* genes was influenced by fermentation parameters and pH inducible promoters (67).

For folate, of the 148 *B. adolescentis* genomes analyzed, 74% contained the full complement of genes known to produce folate *de novo*. There was wide variation in the amount of extracellular folate produced by different strains *in vitro*, from 23 to 180 ng/ml. However, all produced folate, including six that lacked either DHPPP 3.1.3.1 or *pabC*. Other members of the gut microbiota have been identified with missing genes (68), suggesting that there might be redundancy in the folate biosynthetic pathways or alternative metabolic routes that compensate for missing genes. In addition, polyglutamation of folate may limit export from the bacteria and lead to a difference between the total folate produced and the amount of folate released extracellularly (37).

In addition to investigating the prevalence of beneficial traits such as β-galactosidase activity and resistant starch utilization, we examined the broader question of which carbohydrate utilization pathways make *B. adolescentis* unique at the species and strain level. Previous research has shown that *B. adolescentis* specializes in metabolizing plant compounds (12). In addition to the presence of starch gene clusters, most of the tested *B. adolescentis* strains were adept at utilizing soluble starch *in vitro*. Putative arabinoxylan gene clusters were also identified as more common (most strains had two clusters) in *B. adolescentis*, although a third of the strains could not grow on arabinoxylan *in vitro*, suggesting that other enzymes may be necessary to metabolize this polysaccharide. Within *B. adolescentis*, CAZyme profiles separated all 148 strains into one of three different clusters. Characterizing the enzymatic families that distinguish each group should provide insight into the ecological reasons for their similar specialization. Interestingly, these clusters of different metabolic capacity did not correspond with the core gene phylogeny and suggest that some CAZyme genes and clusters are readily lost or gained. Arzamasov et al. (43) also recently showed a lack of concordance between phylogeny and predicted phenotypes within multiple *Bifidobacterium* species, including *B. adolescentis*, *B. breve*, *B. pseudocatenulatum*, and *B. longum*.

Previous reports have shown that *B. adolescentis* is among the most prevalent and abundant bifidobacteria in healthy adults (8, 32, 69, 70). Our analysis here is consistent with these previous findings (71–73) and shows that *B. adolescentis* generally increases in abundance from childhood to middle age, before declining later in life. However, large-scale trends may obscure differences for specific populations, such as people with exceptional longevity whose gut microbiota is enriched in *B. adolescentis* (8, 23). Increased levels of *B. adolescentis* in adults corresponded with the metabolic potential that we identified for *B. adolescentis*, including its ability to utilize lactose and many complex carbohydrates (e.g., FOS, raffinose, resistant starch) at a time of decreasing host lactase activity and increasing consumption of plant fibers (19).

Future investigations into *B. adolescentis* distribution and the prevalence and activity of its beneficial traits would be valuable. Although there are very few sequenced non-human *B. adolescentis* isolates (possibly due to bias in sampling and sequencing), there have been many *B. adolescentis* 16S rRNA genes reported from multiple animals (48–50). This study successfully isolated one non-human *B. adolescentis* strain from a cotton-top tamarin. Analysis of more human and non-human *B. adolescentis* isolates, plus comparator strains that are more closely related (such as *Bifidobacterium ruminantium*) and other species that share the same gastrointestinal niche (such as *B. pseudocatenulatum*) will be valuable to understand the evolution of traits that are beneficial to the host. It will also be important to examine the presence and activity of other potentially beneficial traits that were not examined in this study. For example, *B. adolescentis* are additionally known to produce lactate and acetate (74) and various anti-inflammatory compounds (75), modulate the host immune system (e.g., by exopolysaccharides [76]), and improve the intestinal epithelial cell barrier (3).

In summary, many potentially beneficial traits are widespread across *B. adolescentis*, but variable in their level of activity or production. This work points to the importance of performing both genetic and laboratory testing of potentially beneficial properties, especially under relevant conditions that simulate the *in vivo* environment. Therefore, due to the high abundance of *B. adolescentis* in healthy adults and its multitude of beneficial properties, our data suggest that many strains of *B. adolescentis* are promising candidates for promoting health.

## MATERIALS AND METHODS

### Metagenomic comparison of *Bifidobacterium* through life

To assess relative abundance of *B. adolescentis* in the population, we utilized the curatedMetagenomicData R package v3.1.2 (47). The package includes a curated shotgun metagenomic database of 68 studies containing 20,683 samples from 34 countries between 2013 and 2022. Data were filtered to only include stool samples from healthy individuals that reported age at time of sampling: 5,966 samples from 37 studies from 23 countries (Table S1; ). Besides health, most other metadata was unavailable or not statistically powered, although there were likely differences according to geographic region of origin.

### Isolation and identification of *B. adolescentis* from fecal samples and commercial probiotics

In order to identify a diverse range of *B. adolescentis*, we collected fecal samples from humans as well as domestic, zoo, and wild animals (Table S3). Human samples were collected under Advarra IRB# Pro00059566. Written informed consent was obtained prior to sample collection. Fresh fecal samples from animals were provided by the Lincoln Children’s Zoo (Lincoln, NE). Several wild animal samples were collected from snow in the winter.

Fecal samples were spread on several media, including BSIM, MRS (BD Difco), RCM (BD Difco), BHI (Oxoid), YCFA (77) + 2 g/L glucose, maltose, and cellobiose (78), Rogosa SL (BD Difco), and GOS-RCM (RCM with no agar, starch, or dextrose, plus 0.5% GOS [Shanghai Freemen]). BSIM was prepared by supplementing MRS with 13 g/L agar, 500 mg/L L-cysteine-HCl, 20 mg/L nalidixic acid, 50 mg/L mupirocin, 25 mg/L kanamycin, 50 mg/mL polymyxin B sulfate, 8.5 mg/L iodoacetate, and 25 mg/L 2,3,5-triphenyltetrazolium chloride (79). In addition, we obtained commercially available probiotic products that claimed to contain *B. adolescentis* (Table S4). Commercial probiotic capsules were opened aseptically, suspended in PBS, spread on BSIM agar plates, and incubated in an anaerobic chamber filled with 90% N_2_, 5% CO_2_, and 5% H_2_ gas mix. Isolated colonies were picked from plated fecal samples or commercial products and grown overnight in MRS broth. Overnight cultures were centrifuged for 5 min at 10,000 × *g* and DNA was extracted from pellets using the DNeasy PowerLyzer Microbial kit (Qiagen). DNA was amplified using 16S rRNA gene forward primers 8F (AGAGTTTGATCCTGGCTCAG) or 373F (ACTCCTACGGGAGGCAGCAG) and reverse primers 1391R (GACGGGCGGTGTGTRCA) or 1492R (GGYTACCTTGTTACGACTT). Thermocycling conditions included the following: (i) an initial denaturation step of 30 s at 98°C; (ii) 35 cycles of 10 s at 98°C, 30 s at 51°C or 58°C, and 1  min at 72°C; and (iii) a final extension of 1 min at 72°C. Amplicons were purified using the GeneJET PCR Purification kit (Thermo Fisher) and quantified with a NanoDrop OneC spectrophotometer (Thermo Fisher). The purified PCR products were Sanger sequenced with primers 8F or 373F (MCLAB). Identification of isolates was conducted using NCBI BLASTn and species were assigned based on sequence similarity.

Additionally, qPCR using *B. adolescentis*-specific primers was performed on commercial probiotic products that claimed to contain *B. adolescentis*. DNA was amplified in triplicate using a QuantStudio 3 (Applied Biosystems) and each reaction mixture contained 10 µL of qPCR Master Mix (2× Maxima SYBR Green; Thermo Fisher), 0.3 µM primers (GGATCGGCTGGAGCTTGCTCCG and CCCCGAAGGCTTGCTCCCAGT) (80), 6.5 µL of water, and 2 µL of template DNA for a final volume of 20 µL. Initial denaturation at 95°C for 10 min, 40 cycles at 95°C for 15 s, 64°C for 30 s, and 72°C for 30 s. A standard curve was made using DNA purified from a culture of *B. adolescentis*.

To narrow the isolates that would be tested for potential beneficial traits, genomic DNA was extracted from *B. adolescentis* strains and used as template in PCR targeting the ribosomal RNA internal transcribed spacer as previously described (Bif16S_1525-1543F: GCTGGATCACCTCCTTTCT; 23S_40-23R: CTGTCTGCCAAGGCATCCA) (81). Amplicons were Sanger sequenced (MCLAB) using primer Bif16S_1525-1543F, then analyzed phylogenetically by aligning ITS sequences with MAFFT v7.490 (82) and inferring an approximately-maximum-likelihood phylogenetic tree with FastTree v2.1.10 (83). The tree was visualized with iTOL v6.5.6 and the genomes of diverse, representative strains were chosen for Illumina sequencing.

### Genome sequencing and assembly of *B. adolescentis* isolates

*B. adolescentis* isolates were grown overnight in MRS broth, centrifuged for 5 min at 10,000 × *g* and the DNA extracted using the DNeasy PowerLyzer Microbial kit (Qiagen). Genomic libraries were prepared and 2 × 151 bp reads sequenced on an Illumina NovaSeq X Plus (SeqCenter) using the Illumina 200 Mbp (1.33 M reads) service. Paired-end reads were quality trimmed using Sickle version 1.33 (84) and assembled *de novo* using SPAdes version 3.15 (85). Table S21 shows the number of reads generated per sample and assembly statistics. Coverage was calculated by mapping reads to a complete reference genome (*B. adolescentis* iVS-1) using bwa v0.7 (86) and samtools v1.9 (87).

### *B. adolescentis* genome selection

A total of 892 *B. adolescentis* genomes were available from the NCBI public database as of November 2024. From this list, samples were filtered to (i) exclude atypical genomes (chimeras, contaminated, or other major problems) and (ii) use GCF genome assemblies of high quality (≥95% completeness and ≤4% contamination) resulting in 280 genomes. Additionally, 12 genomes of *B. adolescentis* strains isolated during this study were included in the analyses. The average nucleotide identity (ANI) between strains was calculated with fastANI (v1.32) (88) (range, 97.3–100% ANI). To reduce redundancy from highly similar genomes, a representative from each group (>99.9% ANI) was chosen, prioritizing strains with the most complete genomes and available metadata. This resulted in a total of 148 high-quality, non-redundant *B. adolescentis* genomes (Table S2).

### Genome annotation and phylogeny

Genome annotation was performed using PROKKA with default parameters (89). Annotated protein sequences were used as input for Orthofinder v2.5 (90), from which 1,117 orthogroups were selected to construct a phylogenetic tree. Multiple sequence alignment was conducted with MAFFT v7.490 (82), and an approximately maximum-likelihood phylogenetic tree was inferred with FastTree v.2.1.10 (83) and visualized using iTOL v6.5.6 (https://itol.embl.de). To characterize CAZymes, genomes were annotated against the Carbohydrate Active Enzymes database (CAZy [91]) using dbCAN3 (92). Identified CAZyme domains were further validated with InterProScan v5.52 using Pfam, SUPERFAMILY, and SMART annotations (93). Beta-galactosidase and GABA genes were retrieved based on their Enzyme Commission (EC) number (3.2.1.23 for beta-galactosidase, 4.1.1.15 for *gadB*, and COG0531 for *gadC*). While *gadA* and *gadB* were initially distinguished in *E. coli*, we followed the conventional naming of all homologs as *gadB* to maintain consistency (36, 94). We also examined CAZyme families associated with resistant starch metabolism, including GH13, GH31, GH77, CBM48, CBM25, and CBM26 (63, 95, 96). Validation of CAZyme-resistant starch annotation was performed with InterProScan. The genes (with EC numbers) for *de novo* folate biosynthesis were also examined and included the following: (i) chorismate pathway (six genes): *aroG* 2.5.1.54, *aroB* 4.2.3.4 + *aroK* 2.7.1.71, *aroQ* 4.2.1.10, *aroE* 1.1.1.25, *aroA* 2.5.1.19, and *aroC* 4.2.3.5; (ii) pABA pathway (two genes): *pabA* 2.6.1.85 and *pabC* 4.1.3.38; (iii) DHPP pathway (four genes): *folE* 3.5.4.16, 3.1.3.1, *folQ* 3.6.1.-, and *folB* 4.1.2.25 + *folK* 2.7.6.3; and (iv) THF-polyglutamate pathway (three genes): *folP* 2.5.1.15, *folC* 6.3.2.12, and *folA* 1.5.1.3 (14, 37). A comparative analysis of folate genes was performed with large scale blast score ratio (LS-BSR) pipeline (97) (Table S19), using *B. adolescentis* iVS-1 folate genes as reference. Genes with ≥40% identity were counted as present (common functional similarity cutoff [98]), but since the identity to genes for some comparator strains was slightly lower, we also reported when identities were 30–40%.

### Analysis of genes encoding for carbohydrate metabolism

Carbohydrate profiles for all 148 *B. adolescentis* and 16 comparator strains were assessed using dbCAN3 (92). To evaluate differences in CAZyme substrate family counts between *B. adolescentis* and comparators, we performed differential abundance analysis using DESeq2 (99) and edgeR (100). Raw absolute counts of CAZyme families were obtained from dbCAN annotations and filtered to retain only families with a minimum of five counts across all samples, reducing noise from low-abundance CAZymes. Metadata containing sample group information was incorporated, and counts were normalized using size factors in DESeq2 (poscounts) and TMM normalization in edgeR to ensure robust normalization for sparse data and account for library size differences. Differentially abundant CAZymes were identified based on thresholds of an FDR-adjusted *P*-value < 0.05 and ≥2 copies. Significant CAZyme families common to both methods were extracted for further analysis. Heatmaps were generated using normalized counts of significant CAZyme families to visualize clustering patterns. Principal component analysis (PCA) was performed on variance-stabilized counts. CAZyme grouping was determined using k-means clustering. Final figures were prepared using ggplot2 (101) and R package pheatmap v.1.0.12. To further investigate polysaccharide degradation ability, we used CGC-finder from dbCAN to identify CGCs, consisting of at least one CAZyme, one transporter, one transcriptional regulator, and one signaling transduction protein. Additionally, dbCAN-PUL was used to predict potential experimentally validated substrates for CGCs.

### Carbohydrate utilization

A total of 47 compounds (sources and purity listed in Table S22) were tested for their ability to support growth in or on MRSc (MRS + 0.5 g/L cysteine) without glucose and containing 10 g/L of individual carbohydrates. Thirty-nine carbohydrates were added to broth media, while eight opaque carbohydrates were added to agar plates. Liquid media was inoculated 1:100 from overnight cultures into 96-well polystyrene plates in triplicate. The optical density at 620 nm was monitored with a Tecan Sunrise plate reader using Magellan (v7.3) software. Liquid overnight cultures were also inoculated onto agar plates and growth assessed as no/low, medium, or high growth based on visual growth compared to plates with no carbohydrate (no/low growth). Strains were grown for 72 h at 37°C in an anaerobic chamber.

### Resistant starch utilization

To assess the ability of strains to metabolize a type 2 resistant starch, each strain was grown anaerobically with shaking at 37°C for 48 h in MRS containing 100 mM potassium phosphate buffer (pH 6.8) anad 1% potato starch (Bob’s Red Mill) as the only carbohydrate. After 48 h, the cultures were confirmed to remain above pH 6.0. Samples and uninoculated controls were assayed in duplicate using the Neogen/Megazyme Resistant Starch (Rapid) kit, following the manufacturer’s instructions. This method removes non-resistant starch and simple carbohydrates before converting resistant starch to a red compound that is measured in a spectrophotometer at 510 nm. For the two comparator strains that utilized resistant starch, since there were no previous reports of this capability, the experiment was repeated, and similar values were obtained.

### β-Galactosidase activity

β-Galactosidase assays were conducted as previously described (10). Briefly, active cultures were used to inoculate reduced complex gut media + MOPS (CGMM) supplemented with 1% lactose (100% purity, Loudwolf). CGMM is composed of peptone (0.5 g/L), yeast extract (1 g/L), sodium chloride (1 g/L), magnesium sulfate (0.1 g/L), calcium chloride (0.1 g/L), hemin (0.005 g/L), cysteine (0.5 g/L), bile salts (0.5 g/L), sodium acetate (1.35 g/L), sodium propionate (0.43 g/L), isobutyric acid (0.5 mM), isovaleric acid (0.5 mM), valeric acid (0.5 mM), sodium bicarbonate (4 g/L), potassium phosphate (0.45 g/L each of KH_2_PO_4_ and K_2_HPO_4_), vitamin K1 (1 mg/L), maltose (0.1 g/L), cellobiose (0.1 g/L), inulin (0.1 g/L), arabinogalactan (0.1 g/L), soluble starch (0.1 g/L), and 3-(N-morpholino)propanesulfonic acid (MOPS) buffer pH 7.3 (100 mM). Cells were grown at 37°C for 6 h, harvested by centrifugation, and the pellet was washed and resuspended in Z buffer. Cells were enumerated by dilution plating and activity measured for each of two preparations: directly (whole cell) or lysed (beaten with 0.1 glass beads). Samples were assayed for β-galactosidase activity by addition of ortho-nitrophenyl-β-galactoside (ONPG) solution. After 5 min, the reactions were centrifuged for 3 min, and supernatants were aliquoted in triplicate into 96-well plates with sodium carbonate stop solution. Optical densities were determined in a microplate reader (BioTek Synergy H1). β-Galactosidase activity (Miller units) was determined using the formula 1,000 × (*A*_420_ − 1.75 × *A*_550_)/(time (min) × vol (mL) × CFU correction factor (normalized to 10^7^)). All strains had a density of at least 10^7^ CFU/mL. There was no correlation between cell density and β-galactosidase activity (*r* = −0.07). Cells were grown in duplicate and enzymatic activity was measured in triplicate.

### Gas reduction experiments

Gas experiments were performed following the protocol described in Ramakrishnan et al. (10). Briefly, test strains were grown in lactose with fecal samples from one of two donors. Individual strains were prepared by growing in RCM or MRS (BD Difco). Cells were collected in late log phase, pelleted by centrifugation, resuspended in CGMM containing 7% dimethyl sulfoxide (DMSO) cryoprotectant, and frozen at −80°C. At time of experiment, cells were thawed, added to 2 mL vials with screw caps containing septa composed of PTFE and silicone. Cells were added at equal cell concentrations (10^7^ CFU). Fecal communities (0.5 mL) were added after diluting frozen fecal samples 1:50 in CGMM + lactose. Fecal donations were collected under Advarra IRB# Pro00059566. Experiments were conducted in an anaerobic chamber filled with 90% N_2_, 5% CO_2_, and 5% H_2_ gas mix. After 18 h at 37°C, shaking at 125 RPM, the amount of gas was measured by inserting a needle attached to a glass syringe through the septum. Change in gas was calculated by the formula 100 × [(mean of no *B. adolescentis* control + DMSO) − individual test sample]/(mean of no *B. adolescentis* control + DMSO).

### GABA production

To determine GABA production, strains were grown 24–48 h on MRS or RCM agar plates, then inoculated into 15 mL conical tubes of pre-reduced MRS + 1% glutamic acid, pH 5.6, based on optimization in previous studies (13, 102). Cultures were grown for 48 h, centrifuged, and the supernatants collected. Five milliliters of supernatants or standards containing media + GABA was lyophilized. An aliquot of the powder was submitted to the University of Nebraska-Lincoln Proteomics and Metabolomics facility, where amino acids were extracted using methanol and chloroform, derivatized using AccQ-Tag reagent, then analyzed by Free Amino Acids Ultra Performance Liquid Chromatography (Waters) following standard protocols.

### Folate production

Extracellular folate concentration was measured with a bioassay using Folic Acid Casei medium (FAC; HIMEDIA) and growth of folate-dependent *Lactobacillus rhamnosus* (previously *casei*) ATCC 7469. Test strains were inoculated into MRSc broth, incubated anaerobically for 24 h at 37°C, centrifuged, and supernatants were filter sterilized. Each strain was tested in at least triplicate. Samples were stored at −20°C until analysis. To prepare *L. rhamnosus*, the strain was inoculated into MRSc broth and incubated aerobically overnight at 37°C, and 1 mL culture was centrifuged. The pellet was washed three times in FAC, then inoculated 1:1,000 into fresh FAC and incubated at 37°C for 18–24 h.

For folate measurements, 20 µL of the test supernatants, standards, or negative control were added to 10 mL 0.5× FAC and autoclaved for 5 min. After cooling, 10 µL of *L. rhamnosus* culture was added. Tubes were incubated aerobically at 37°C for 18–24 h with shaking (120 rpm). Tubes were then vortexed, and growth was determined by measuring optical density at 600 nm using the uninoculated negative control as the blank. Control *L. rhamnosus* grown on folate-containing medium had very similar growth curves ± bacterial supernatants, suggesting antimicrobials did not interfere with the assay.

### Identification of genes related to safety and production of antimicrobial compounds

Antibiotic resistance genes were identified using the Comprehensive Antibiotic Resistance Database Resistance Gene Identifier (103). Virulence genes were analyzed with VFDB (104) and VirulenceFinder 2.0 (105). Potential bacteriocin synthesis gene clusters were identified using BAGEL4 (106). The presence of other secondary metabolite biosynthetic gene clusters was evaluated using antiSMASH 7.0 (107).

## Data Availability

The raw genome data, assembled genome scaffolds, and Sanger sequences have been deposited under the NCBI BioProject ID PRJNA1203657 and PV094590–PV094617. R code used to generate figures and calculate statistics is available at https://github.com/auchtung/VanHaute2025.

## SUPPLEMENTAL MATERIAL

Supplemental Figures msphere.00673-25-s0001.docx Figures S1 to S7.

Supplemental Material msphere.00673-25-s0002.docx Supplemental text.

Supplemental Tables msphere.00673-25-s0003.xlsx Tables S1 to S25.

ASM does not own the copyrights to Supplemental Material that may be linked to, or accessed through, an article. The authors have granted ASM a non-exclusive, world-wide license to publish the Supplemental Material files. Please contact the corresponding author directly for reuse.
