# Supplementary material for: Comparative genomic and phenotypic analysis of potential beneficial properties of Bifidobacterium adolescentis
Source: mSphere. 2025 Nov 4;10(11):e00673-25. doi: 10.1128/msphere.00673-25 (PMC12646000; doi:10.1128/msphere.00673-25)
Supplement: Supplemental Figures — Figures S1 to S7. [file msphere.00673-25-s0001.docx]

**SUPPLEMENTAL FIGURES S1-S7.**


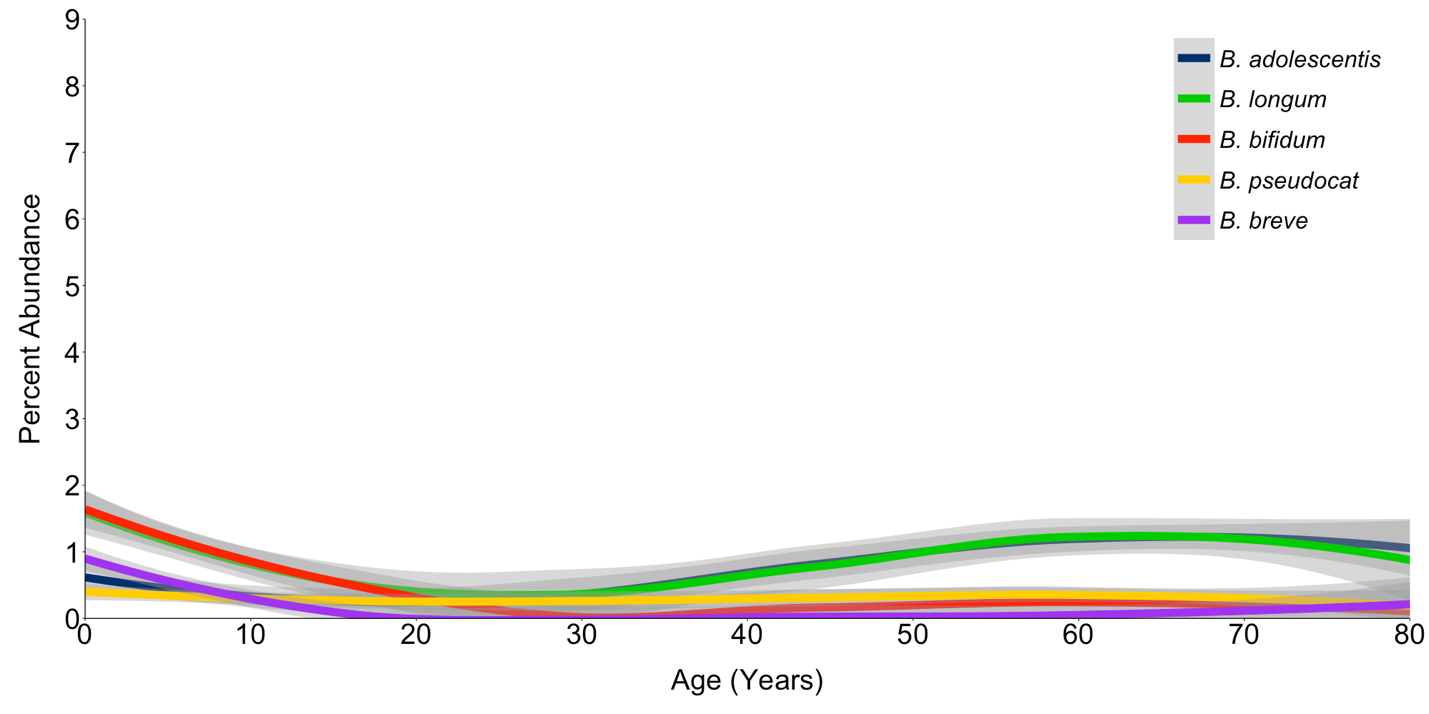


**Fig. S1. Abundance of *Bifidobacterium* in unhealthy individuals by age.** LOESS fit for abundance of the five most common *Bifidobacterium* species in a curated database of shotgun metagenomic sequences from stool samples (n = 2,976 samples). Shaded regions represent 95% confidence intervals. Full data listed in Table S1.

**
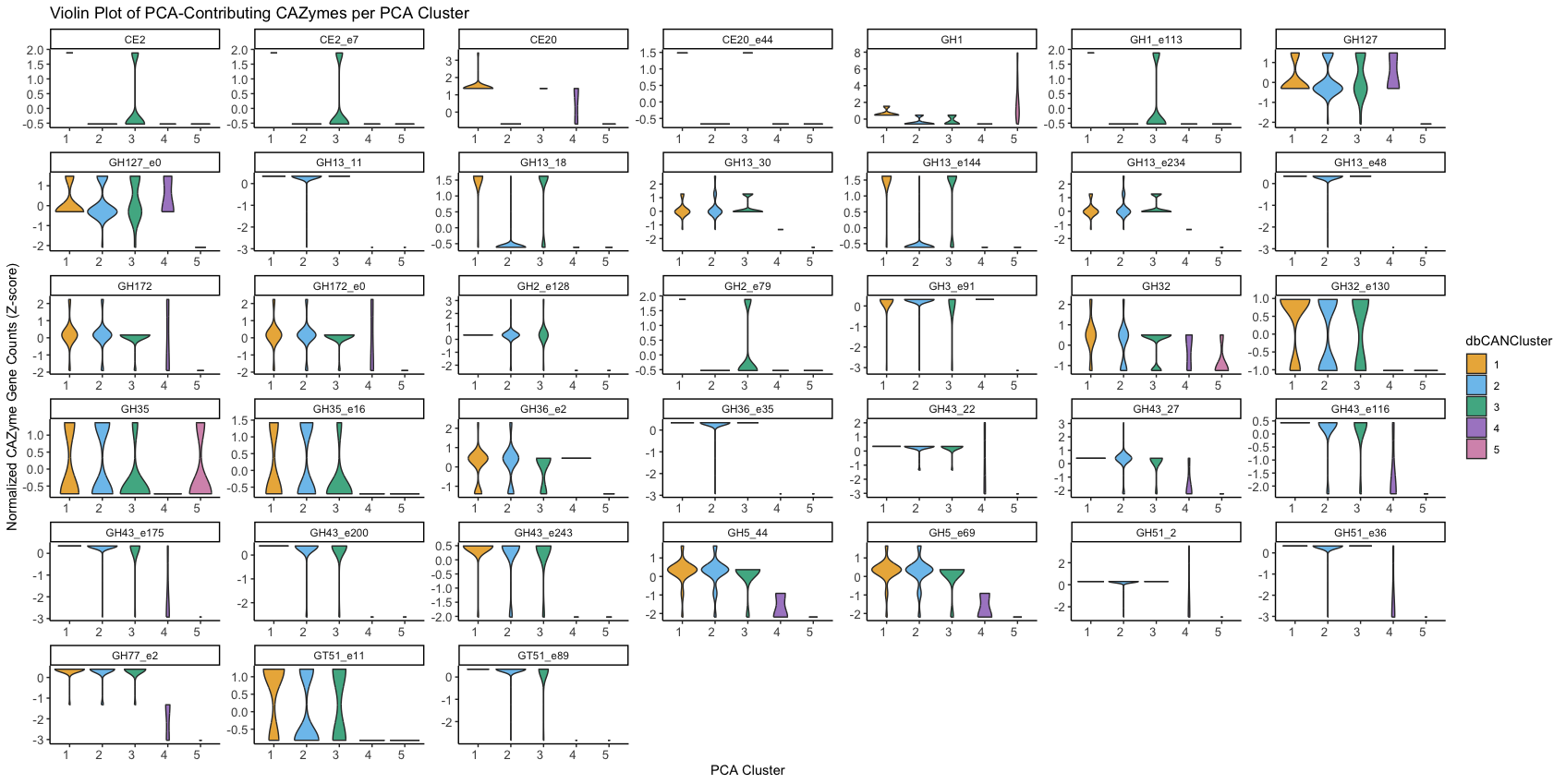
**

**Fig. S2. Contribution of CAZyme families to clustering of strains.** Violin plot displaying the density distribution of normalized CAZyme counts for K-means clusters in *B. adolescentis* (clusters 1, 2, 3) and comparator groups (clusters 4, 5). Wider sections of each violin indicate higher sample density. GH, glycoside hydrolase. GT, glycosyl transferase. CE, carbohydrate esterase. Full data listed in Table S8.

**
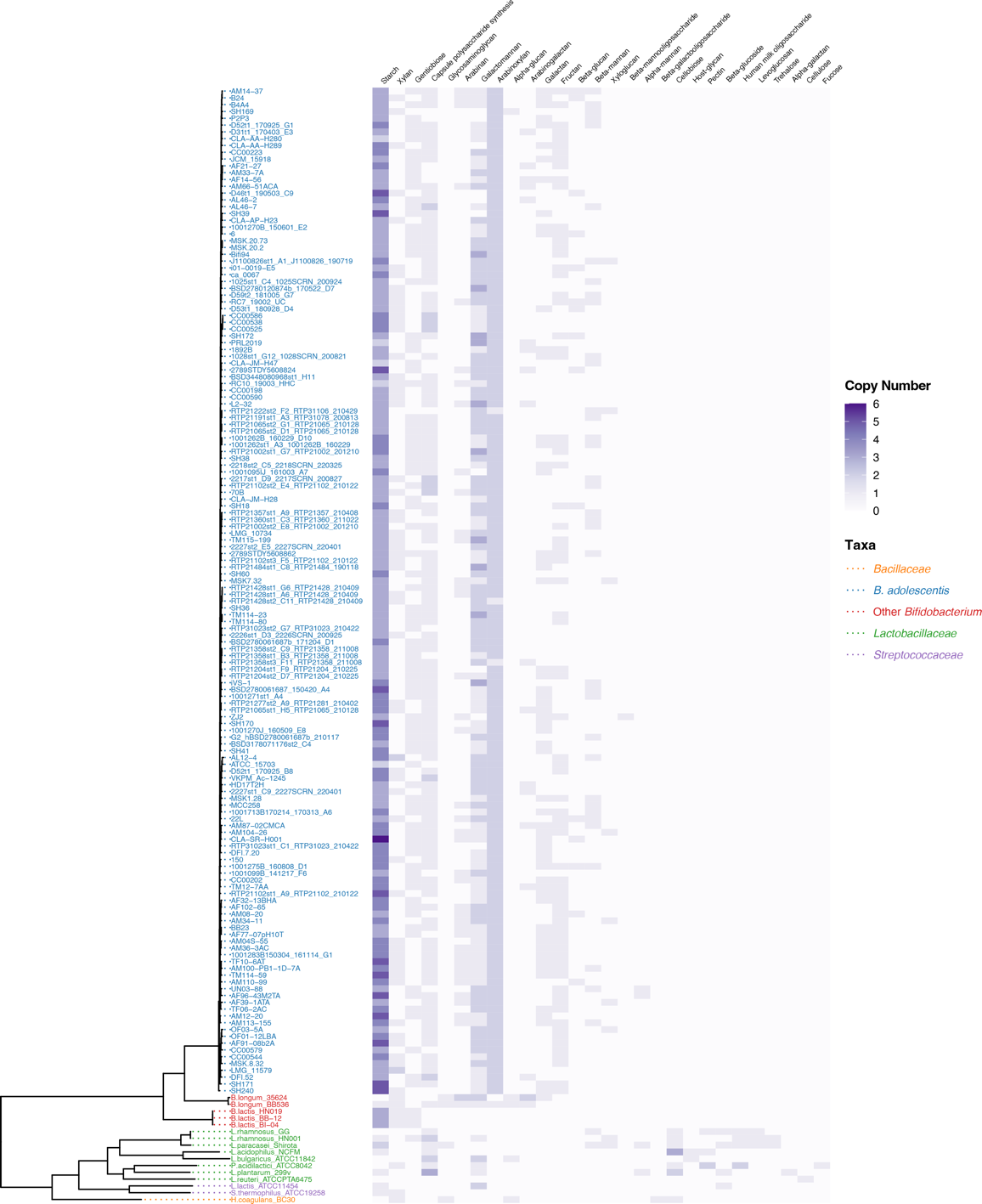
**

**Fig. S3.** **Heatmap of *B. adolescentis* CAZyme gene clusters.** Functionally characterized CAZyme gene clusters with copy number ≥2 and significantly different (FDR-adjusted p < 0.05) between *B. adolescentis* and comparator strains by analysis with both DESeq2 and edgeR. Normalized data were clustered by Euclidean distance using Ward’s method. The heatmap is arranged according to phylogeny, as predicted in Orthofinder with MAFFT and FastTree analysis of 1,117 single-copy orthogroups. Full data and taxonomic names listed in Table S9.

**Fig. S4. Gas reduction in *B. adolescentis* strains.** Change in gas produced by two fecal communities in CGMM + lactose, assessed in triplicate. Displayed values are relative to communities with no *B. adolescentis* added. Each shape is a different fecal donor. Strains are ordered from highest to lowest β-galactosidase activity as previously assessed. Box plots show the interquartile range (IQR; boxes), median (line), and 1.5 IQR (whiskers). For comparisons with the no *B. adolescentis* control, p < 0.005 by Mann-Whitney-Wilcoxon test for all strains, except for SH41 (p = 0.17). See Table S16 for full data.

**
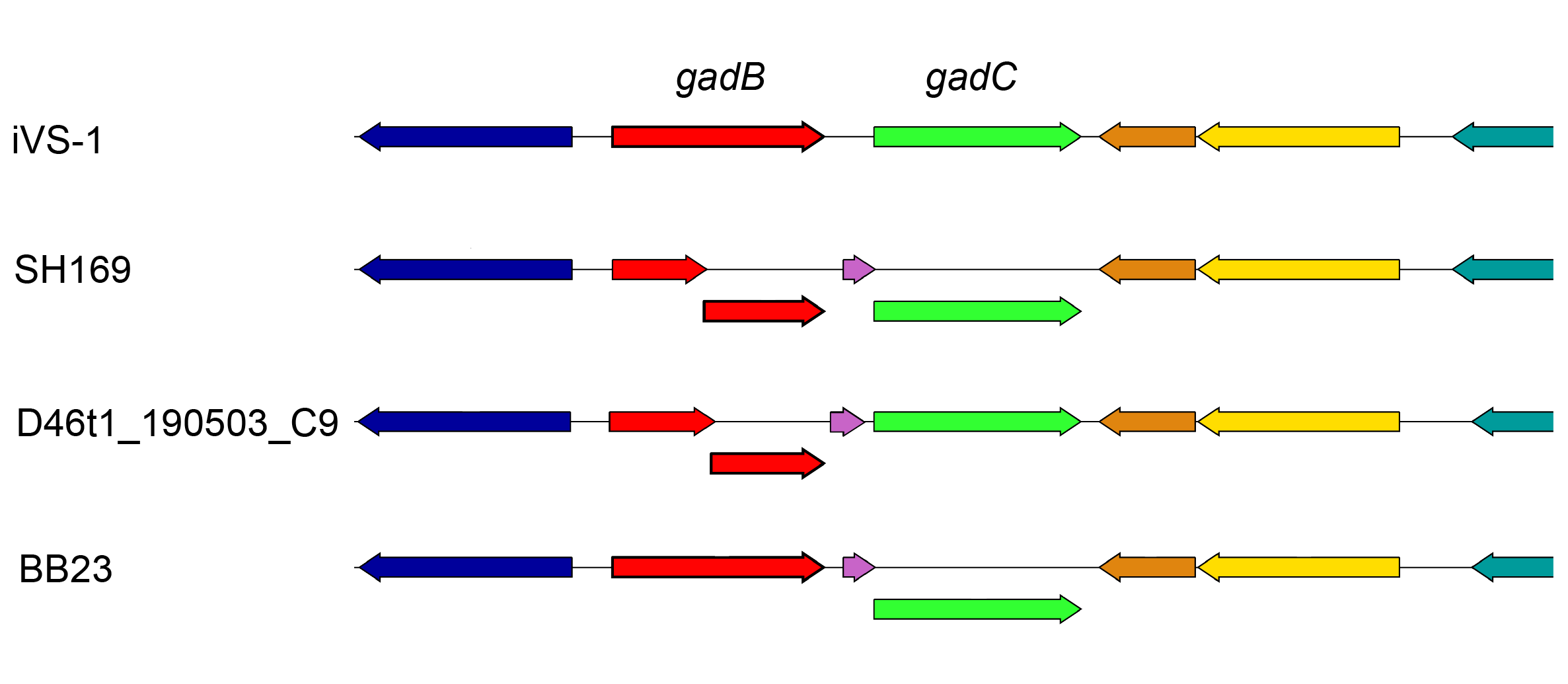
**

**Fig. S5. Synteny for the GABA gene cluster in *B. adolescentis***. The primary genes for GABA production are *gadB* (red) and *gadC* (green). Upstream is a gene for a hypothetical protein (blue), and downstream are genes for peptide deformylase (brown), phosphoglucosamine mutase (yellow), and membrane alanine aminopeptidase N (aqua). SH169, D46t1_190503_C9, and BB23 possess short hypothetical proteins (purple) that are not in the other 145 strains (iVS‑1 shown as a representative). In addition, *gadB* in SH169 and D46t1_190503_C9 are split into two separate genes.





**Fig. S6. Abundance of *Bifidobacterium* in healthy individuals by age and number of samples.** A) LOESS fit for abundance of the five most common *Bifidobacterium* species in a curated database of shotgun metagenomic sequences from stool samples (n = 5,966 samples). Shaded regions represent 95% confidence intervals. B) Number of samples analyzed by age. Full data listed in Table S1.

**
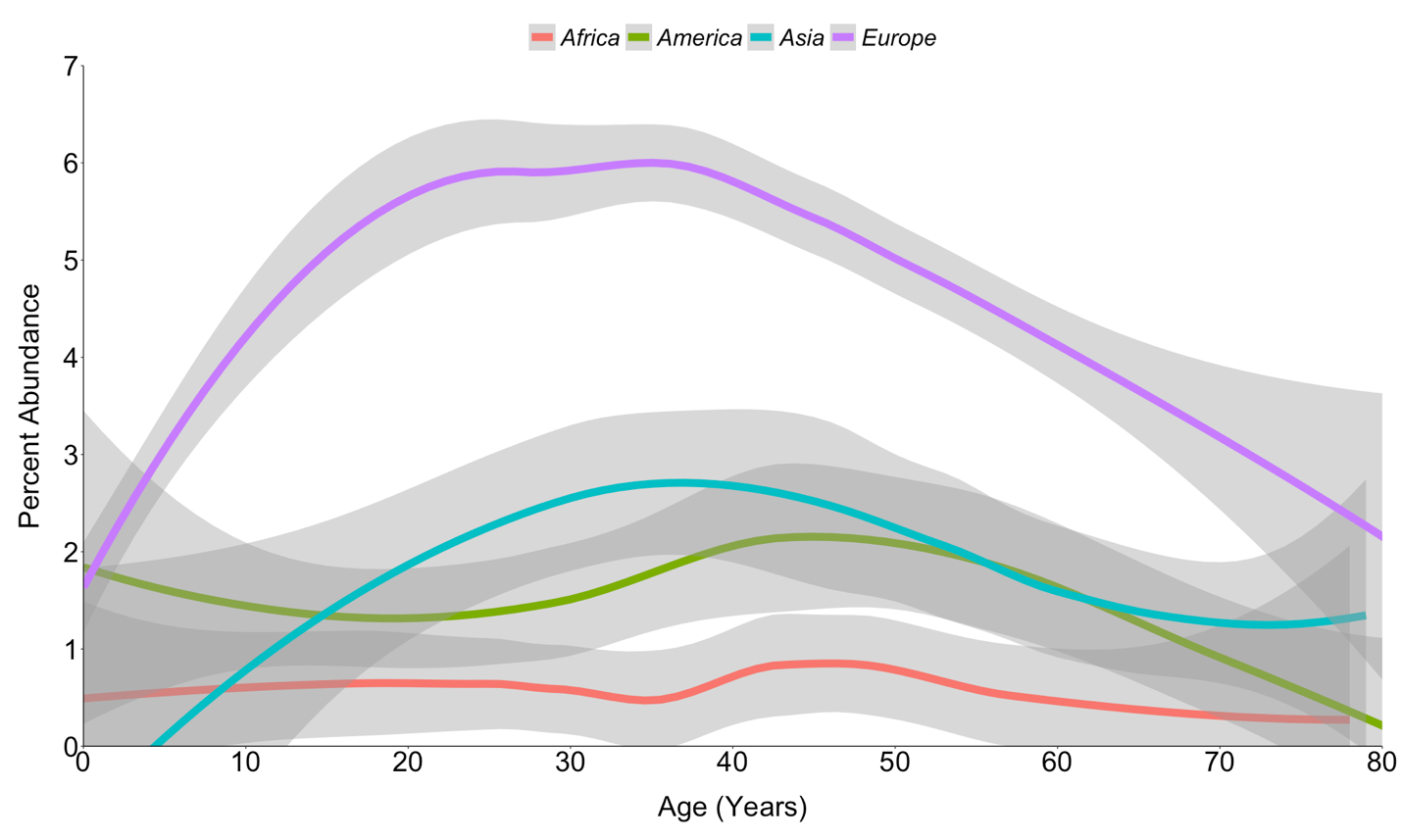
Fig. S7. Abundance of *B.* *adolescentis* in healthy individuals by geographical region.** LOESS fit for abundance of *B.* *adolescentis* in a curated database of shotgun metagenomic sequences from stool samples of four African countries (n = 358 samples), three Asian countries (n = 436 samples), twelve European countries (n = 3,891 samples), and three American countries (n = 1,109 samples). Shaded regions represent 95% confidence intervals. Full data listed in Table S1.
