# Supplementary material for: Comparative genomic and phenotypic analysis of potential beneficial properties of Bifidobacterium adolescentis
Source: mSphere. 2025 Nov 4;10(11):e00673-25. doi: 10.1128/msphere.00673-25 (PMC12646000; doi:10.1128/msphere.00673-25)
Supplement: Supplemental Material — Supplemental text. [file msphere.00673-25-s0002.docx]

**SUPPLEMENTAL TEXT**

Identification of genes for production of antimicrobial compounds

We investigated the potential for *B. adolescentis* to inhibit possible pathogenic bacteria through production of bacteriocins or other secondary metabolites by searching the BAGEL and antiSMASH databases. Only six *B. adolescentis* genomes had genes associated with a bacteriocin, propionicin SM1. Five other genomes had hits to secondary metabolite biosynthetic gene clusters (SMBGCs) coding for non-ribosomal peptide synthetase (NRPS)-like enzymes (Table S23). This class has diverse functions that may or may not be antimicrobial. Conversely, 10/16 non-*B. adolescentis* genomes contained at least one putative bacteriocin gene and hits for SMBGCs.

That genes for producing antimicrobials were identified in only 7% of *B. adolescentis* strains is consistent with other studies suggesting that bacteriocins are common among non-bifidobacterial lactic acid bacteria (1, 2), but uncommon in most *Bifidobacterium* species, and particularly rare in *B. adolescentis* (3). The bacteriocin genes from *B. adolescentis* strains were similar to one characterized from *Propionibacterium jensenii* (4), though homologues have also been identified in multiple *Bifidobacterium* species (5, 6). Some *Bifidobacterium* strains also contained a non-ribosomal peptide synthetase. This antimicrobial class has many different categories, and significant work is required to define the range of activity *in vitro* and *in vivo* (7). This study was limited from further examination of antimicrobial production as none of the *B. adolescentis* strains in our collection contained the relevant genes.

Genomic safety assessment
 Potential safety-related genes encoding antibiotic resistance or virulence factors were identified for all strains using the CARD, VFDB, and VirulenceFinder databases. For the 148 *B. adolescentis* genomes, all strains shared an allele of *rpoB* associated with resistance to rifamycin (rifamycin-resistant beta-subunit of RNA polymerase) with >99% identity (Table S24). Additionally, some isolates possessed genes conferring resistance to tetracycline (*tetO/tetW*; 25/148), trimethoprim (*dfrF*; 5/148), macrolides (*ermX*; 8/148), or chloramphenicol (*catA1*; 1/148)(Fig. 2). These strains were distributed across all five lineages with no phylogenetic association, suggesting these genes had been acquired on multiple occasions. For the non-*B. adolescentis* comparator strains, genes putatively encoding resistance to lincosamides, quaternary ammonium compounds/disinfecting agents, rifamycin, fluoroquinolones, tetracycline, and/or vancomycin were observed in 12/16 strains. Genes for virulence factors were not detected in any *B. adolescentis* or comparator strains (Table S25), although activity was not tested *in vitro*.

In summary, most (77%) *B. adolescentis* strains contained no antibiotic resistance genes in their genomes, however, nearly one fourth of the strains contained one or more genes for antibiotic resistance. *Bifidobacterium* are considered less likely than lactobacilli and enterococci to transfer antibiotic resistance genes (8), although high levels of resistance have been identified in some *B. adolescentis* strains (9).

**REFERENCES**

1. Gontijo MTP, Silva J de S, Vidigal PMP, Martin JGP. 2020. Phylogenetic distribution of the bacteriocin repertoire of lactic acid bacteria species associated with artisanal cheese. Food Research International 128:108783.

2. Zhang D, Zhang J, Kalimuthu S, Liu J, Song Z-M, He B, Cai P, Zhong Z, Feng C, Neelakantan P, Li Y-X. 2023. A systematically biosynthetic investigation of lactic acid bacteria reveals diverse antagonistic bacteriocins that potentially shape the human microbiome. Microbiome 11:91.

3. Yu D, Pei Z, Chen Y, Wang H, Xiao Y, Zhang H, Chen W, Lu W. 2023. Bifidobacterium longum subsp. infantis as widespread bacteriocin gene clusters carrier stands out among the Bifidobacterium. Appl Environ Microbiol 89:e0097923.

4. Miescher S, Stierli MP, Teuber M, Meile L. 2000. Propionicin SM1, a Bacteriocin from Propionibacterium jensenii DF1: Isolation and Characterization of the Protein and its Gene. Syst Appl Microbiol 23:174–184.

5. Lu W, Pei Z, Zang M, Lee Y, Zhao J, Chen W, Wang H, Zhang H. 2021. Comparative Genomic Analysis of Bifidobacterium bifidum Strains Isolated from Different Niches. Genes (Basel) 12:1504.

6. Bosselaar S, Dhelin L, Dautel E, Titecat M, Duthoy S, Stelmaszczyk M, Delory N, Violante MDS, Machuron F, Ait-Abderrahim H, Desreumaux P, Foligné B, Monnet C. 2024. Taxonomic and phenotypic analysis of bifidobacteria isolated from IBD patients as potential probiotic strains. BMC Microbiol 24:233.

7. Martínez-Núñez MA, y López VEL. 2016. Nonribosomal peptides synthetases and their applications in industry. Sustainable Chemical Processes 4:1–8.

8. Tarracchini C, Viglioli M, Lugli GA, Mancabelli L, Fontana F, Alessandri G, Turroni F, Ventura M, Milani C. 2022. The Integrated Probiotic Database: a genomic compendium of bifidobacterial health-promoting strains. Microbiome Research Reports 1:9.

9. Li W, Liang H, He W, Gao X, Wu Z, Hu T, Lin X, Wang M, Zhong Y, Zhang H, Ge L, Jin X, Xiao L, Zou Y. 2024. Genomic and functional diversity of cultivated Bifidobacterium from human gut microbiota. Heliyon 10:e27270.
